# 

**Published:** 1890-04-05

**Authors:** 


					1.
4> Vol.
SriPril 5th, 1890.
?' v01? Vm.?April 5th, 1890.
Post Office as a Newspaper for
?on in the United Kingdom ar>fl aw? a
Per Annnm, 6s. 6d.; post free. //?
Monthly Parts, 6d.; post free 8df s
Ditto per Annum 8s. post free.!/"" ? )
vino per Annum as. post iree.j^y i ^
I
A WEEKLY JOURNAL OF
Sttietttt, /Ifttirtctm, Iftttrstitg aittr (^ijtlantljfopS

				

